# Japan Environment and Children’s Study: backgrounds, activities, and future directions in global perspectives

**DOI:** 10.1186/s12199-017-0667-y

**Published:** 2017-07-14

**Authors:** Kazue Ishitsuka, Shoji F. Nakayama, Reiko Kishi, Chisato Mori, Zentaro Yamagata, Yukihiro Ohya, Toshihiro Kawamoto, Michihiro Kamijima

**Affiliations:** 1 Department of Medical Subspecialties, National Center for Child Health and Development, 2-10-1 Okura, Setagaya-ku, Tokyo, 157-8535 Japan; 2 Centre for Health and Environmental Risk Research, National Institute for Environmental Studies, 16-2 Onogawa, Tsukuba, 305-8506 Japan; 3 Center for Environmental and Health Sciences, Hokkaido University, North-12, West-7, Kita-ku, Sapporo, 060-0812 Japan; 4 Center for Preventive Medical Sciences, Chiba University, 1-8-1 Inohana, Chuo-ku, 260-0856 Chiba Japan; 5 Department of Health Sciences, Graduate School of Medicine Sciences, University of Yamanashi, 1110 Shimokato, Chuo, 409-3898 Japan; 6 Department of Environmental Health, University of Occupational and Environmental Health, 1-1 Iseigaoka, Yahatanishi-ku, Kitakyushu, 807-0804 Japan; 7 Department of Occupational and Environmental Health, Nagoya City University Graduate School of Medical Sciences, 1 Kawasumi, Mizuho-cho, Mizuho-ku, Nagoya, 467-8601 Japan

**Keywords:** Children’s environmental health, Birth cohort study, Environmental policy, Chemical exposure, International collaboration, Japan Environment and Children’s study (JECS)

## Abstract

There is worldwide concern about the effects of environmental factors on children’s health and development. The Miami Declaration was signed at the G8 Environment Ministers Meeting in 1997 to promote children’s environmental health research. The following ministerial meetings continued to emphasize the need to foster children’s research. In response to such a worldwide movement, the Ministry of the Environment, Japan (MOE), launched a nationwide birth cohort study with 100,000 pairs of mothers and children, namely, the Japan Environment and Children’s Study (JECS), in 2010. Other countries have also started or planned large-scale studies focusing on children’s environmental health issues. The MOE initiated dialogue among those countries and groups to discuss and share the various processes, protocols, knowledge, and techniques for future harmonization and data pooling among such studies. The MOE formed the JECS International Liaison Committee in 2011, which plays a primary role in promoting the international collaboration between JECS and the other children’s environmental health research projects and partnership with other countries. This review article aims to present activities that JECS has developed. As one of the committee’s activities, a workshop and four international symposia were held between 2011 and 2015 in Japan. In these conferences, international researchers and government officials, including those from the World Health Organization, have made presentations on their own birth cohort studies and health policies. In 2015, the MOE hosted the International Advisory Board meeting and received constructive comments and recommendations from the board. JECS is a founding member of the Environment and Child Health International Birth Cohort Group, and has discussed harmonization of exposure and outcome measurements with member parties, which will make it possible to compare and further combine data from different studies, considering the diversity in the measurements of variables between the studies. JECS is expected to contribute to the international environmental health research community and policy-making. More international collaboration would enhance our understanding of the possible environmental causes of diseases and disabilities.

## Background

Exposure to harmful environment in a prenatal period and early lifetime can affect health in both childhood and adulthood [1–4]. The fetus and young children are vulnerable to environmental hazards resulting in serious health problems or altering developmental processes [5]. World Health Organization (WHO) reported that around 20% of global deaths among children under 5 years old were due to modifiable environmental factors [6].

Based on the growing concern about environmental impacts on children’s health, the Miami Declaration on Children’s Environmental Health was signed at the G8 Environment Ministers Meeting in 1997. The Miami Declaration expressed the commitment of the member countries to children’s environmental health, including concerted actions in policy areas and consideration of child health in risk assessments [7]. The following ministerial meetings continued to emphasize the need of fostering children’s research. Especially in 2009, the Japanese Environment Minister and the U.S. Environmental Protection Agency Administrator jointly addressed the G8 Environment Ministers Meeting in Syracuse, Italy, regarding the need for international collaboration on research related to children’s health and the environment, including the impacts of chemicals and heavy metals, the effects of climate change, and improving the knowledge and building the capacity of all professionals involved in children’s environmental health issues.

In Japan, the Ministry of the Environment (MOE) set up an Advisory Commission for Children’s Environmental Health in 2005 to discuss future efforts for children’s environmental health. In 2006, the Commission proposed several activities including MOE-funded key research projects. In light of the commission’s proposition, MOE set up the Advisory Committee of the Epidemiological Research on Children’s Environmental Health in 2007, which recommended the commencement of a large-scale birth cohort study to evaluate associations between environmental chemical exposure and children’s health [8]. Following the recommendation, study protocol planning started under the Working Group of the Epidemiological Research for Children’s Environmental Health in 2008. In April 2010, the Japan Environment and Children’s Study (JECS), a nationwide and government-funded birth cohort study, was launched aiming to evaluate the associations between the environment and children’s health and development. In the JECS, 100,000 pregnant women were enrolled between 2011 and 2014, with their children to be followed up until they reach 13 years of age [9]. In other parts of the world, such nationwide large-scale birth cohort studies are being conducted or have been planned to examine associations between the environment and children’s health. In Europe, for example, two Nordic countries started large-scale birth cohort studies, the Danish National Birth Cohort (DNBC) and the Norwegian Mother and Child Cohort Study (MoBa), in the late 1990s [10, 11]. Both the DNBC and MoBa have recruited more than 100,000 pregnant women [10, 11]. The French Longitudinal Study of Children (ELFE) with 20,000 participating children started an initial enrollment interview of mothers at the child’s birth in 2011, the same year that the JECS started recruitment of pregnant women [12]. The United States of America tried to launch a nationwide birth cohort study named the National Children’s Study with 100,000 participating children, which was canceled in 2014 after 5000 children had been recruited in pilot studies [13, 14] and subsequently replaced with the Environmental influences on Child Health Outcomes (ECHO) program that is aiming to track 100,000 children of existing cohort studies [15]. In Asia, Korea has started a nationwide cohort study named the Korean Children’s Environmental Health Study (KoCHENS) in 2015, in which 100,000 pregnant women will be enrolled [16].

Individual cohort studies may be able to uncover environmental risk factors on children’s health on their own. Moreover, research collaboration between such studies would have benefits in obtaining a large sample size with increased statistical power to enable investigation of the environmental causes of rare diseases. In addition, the international data pooling may allow one to observe more diverse exposures and outcomes reflecting regional, cultural, and ethnic differences among countries [17].

Here, we report international collaboration that JECS has developed, including the International Symposia and International Advisory Board (IAB) meeting.

## JECS International Symposia and launch of the JECS International Liaison Committee

Table 1 shows the JECS international workshop and symposia held by MOE. All the conferences were open to the public free of charge. Participants included researchers, citizens, and the media. The first conferences, which were named the JECS Workshop on International Linkage and JECS International Symposium, were held in Tokyo in February 2011. Affiliations of the conference participants included the WHO, the United Nations Environment Programme, the DNBC, and the National Children’s Study in the United States of America. They recognized the importance of coordination and cooperation between countries and international organizations to enable data pooling and comparison of data from large-scale cohorts in different countries. A recommendation was made to establish an international working group on such harmonization.

**Table 1:** JECS international symposia

Conference title (date)	Venue	Co-sponsor	Country or organization of speakers/panelists	Titles of sessions/presentations
Japan Environment and Children’s Study (JECS) Workshop on International Linkage (2–3 Feb. 2011/workshop was held for researchers)	Tokyo		Japan Denmark Korea United States of America	Session 1: planning large-scale birth cohort studies (five speakers)
			Japan United States of America	Session 2: environmental exposure assessment in cohort studies (two speakers)
			Japan United States of America	Session 3: for what unexpected events must one prepare in site operations? (four speakers)
			Japan	Session 4: follow-up activities in birth cohort studies (one speaker)
			Japan	Session 5: outcome and measurements (one speaker)
			Japan	Session 6: expectation for birth cohort studies (one speaker)
			Australia WHO UNEP	Session 7: international liaison of national cohort studies (three speakers)
JECS International Symposium (4 Feb. 2011/open to the general public)	Tokyo		Japan	Outline of Japan Environment and Children’s Study focusing on the effects of environmental chemicals
			Denmark	The Danish National Birth Cohort
			Korea	Mothers and Children’s Environmental Health Study
			United States of America	The National Children’s Study
			WHO	Coordinating and harmonizing long-term cohort studies of children’s environmental health
			UNEP	An evaluation of environmental health risks and risk management strategies among children in developing countries
			International Childhood Cancer Cohort Consortium (I4C), Australia	International Childhood Cancer Cohort Consortium initiative
			Japan	International linkage in JECS
JECS International Symposium in Kitakyushu, Japan (28 Feb. 2012)	Kitakyushu	University of Occupational and Environmental Health	Japan	Japan Environment and Children’s Study
			United States of America	National Children’s Study USA
			Germany	Concept of a birth cohort study as contribution to health-related environmental monitoring in Germany
			WHO	Coordination and harmonization of the next generation of large-scale birth cohorts
			Japan	Report from WHO Working Group for Coordination of the Next Generation of Large-scale Birth Cohorts
			Japan	Progress in Japan Environment and Children’s Study
			Japan	Current progress of the “Hokkaido Cohort Studies on Environment and Children: congenital abnormality, development, and allergy”
			Japan	A study of child development in northeast Japan
			Japan	Pilot studies for JECS
			Japan	Expectations for JECS
JECS International Symposium, Nagoya, Japan (15 Nov. 2013)	Nagoya	Nagoya City University	Japan	Background to international linkage
			United States of America	U.S. National Children’s Study
			Germany	German activities
			China	Shanghai Birth Cohort
			French	French National Birth Cohort
			United States of America	Report from the working group related to the international collaboration in large-scale birth cohort studies
			Japan	Roadmap of Japan Environment and Children’s Study (JECS) for the future
			Japan	What has been proved and what will be proved in JECS
			Japan United States of America	Expectations for JECS (four speakers)
Fourth JECS International Symposium (15 Dec. 2015)	Tokyo	Chiba University	WHO	Recent global environmental change and children’s health
			Japan	Preliminary results of the Japan Environment and Children’s Study (JECS)
			Norway	The Norwegian Mother and Child Cohort Study
			Denmark	An overview of the Danish National Birth Cohort
			Japan	Current status of birth cohort studies in Asia
			Norway Denmark United States of America Japan	Panel discussion: Promoting birth cohort studies: from the perspective of information dissemination (four panelists)

Soon after, the MOE launched the JECS International Liaison Committee with the following main aims: (a) Collecting information about birth cohort studies conducted outside Japan and methods for measurements of chemical exposure and outcomes relevant to JECS, (b) disseminating information about the study design, methods, and results of JECS to international scientific communities, and (c) international collaboration, including harmonization of study protocols and pooled analysis [18]. In order to collect and share information and opinions, members of the International Liaison Committee attended the scientific conferences (Table 2) and international working group meetings described later. In 2016, the International Liaison Committee also launched a Travel Grant for Young JECS Researchers to train and educate them and thereby to sustain JECS in the decade to come.

**Table 2:** Examples of conference presentations relating to activities of JECS International Liaison Committee

Year	Conferences	Type of presentation	Presenting authors	Titles of presentations
2011	ISES/ISEE^a^	Poster	Mori K	Japan Environment and Children’s Study - Pilot Study and Research Launch
		Poster	Toda E	International Coordination in Birth Cohorts
2013	ISES/ISEE/ISIAQ^b^	Symposium	Nakayama SF	Japan Environment and Children’s Study
2014	ISES	Symposium	Nakayama SF	On-going harmonization of exposure questionnaires among large-scale birth cohort studies
		Symposium	Nakayama SF	Background on the Environment and Child Health International Birth Cohort Group
		Symposium	Nakayama SF	Harmonization of QA/QC measures among large-scale children’s environmental studies
2015	ISES	Symposium	Nakayama SF	Preliminary biomonitoring data from the Japan Environment and Children’s Study
2016	ISES	Symposium	Nakayama SF	Development of personal sampling devices and chemical screening methods for large-scale epidemiology and human biomonitoring studies
		Symposium	Nakayama SF	Biomonitoring as part of exposome measurement in Japan Environment and Children’s Study
		Symposium	Nakayama SF	We do exposome as much as we can: Japan Environment and Children’s Study
	PPTOX V	Symposium	Kamijima M	Present status and preliminary results of the Japan Environment and Children’s Study (JECS)
	ISEE	Poster	Kanatani K	Effect modifiers of desert dust exposure to allergic symptoms–From an adjunct study of Japan Environment and Children Study (JECS)^c^

*Abbreviations*: *ISEE* International Society for Environmental Epidemiology, *ISES* International Society of Exposure Science, *ISIAQ* International Society of Indoor Air Quality and Climate, *PPTOX V* Fifth Conference on Prenatal Programming and Toxicity, *SOT* Society of Toxicology

^a^Joint meeting of ISES and ISEE

^b^ISES, ISEE and ISIAQ hosted the joint meeting

^c^This presentation was supported by the Travel Grant for Young JECS Researchers

The second JECS International Symposium was held in Kitakyushu in 2012. Researchers in the environmental health field made presentations about their own birth cohort studies and children’s environmental health. The third JECS International Symposium was held in Nagoya in 2013. MOE introduced the background and history of an international collaborative activity described later, which was commenced under the initiatives of the WHO and research organizations of the United States of America, Germany, and Japan.

The fourth JECS International Symposium was held in Tokyo in 2015. The speakers and panelists discussed the current status and future of JECS and children’s environmental health in the world. First, the Director of the Department of Public Health, Environmental and Social Determinants of Health of WHO spoke about “Recent Global Environmental Change and Children’s Health.” She mentioned new emerging environmental exposures, including global climate change, ozone depletion, and electronic waste. The environmental burden of diseases in children was the highest in the poorest countries. The JECS results are expected to help global heath communities understand the impacts of the environment on children, and also assist government administrators in developing countermeasures to protect children and develop prevention strategies from adverse effects. Second, the principal investigator of JECS reported that the response rate of follow-up questionnaires was still high in the study at the present time. He also showed the recent results of JECS [19]. Researchers of the DNBC and MoBa also reported the current status of their cohort studies and scientific results.

## International Advisory Board

MOE also held the first IAB meeting on 14 December 2015 [20]. The purpose of the meeting was to receive suggestions and to advice on the design, protocol, and procedures of JECS from international experts involved in environmental health and birth cohort studies in Europe, the United States of America and Asia. Table 3 lists members of the IAB.

**Table 3:** International Advisory Board members [20]

Name	Affiliation
Marie-Aline Charles	Ined-Inserm Elfe, France
Andre Conrad	Toxicology, Health-related Environmental Monitoring, German Environment Agency, Germany
Ruth A Etzel	Office of Children’s Health Protection, Environmental Protection Agency, United States of America
Per Magnus	Norwegian Institute of Public Health, Norway
Mads Melbye	Management Board, Statens Serum Institut, Denmark
Maria P Neira	Department of Public Health, Environmental and Social Determinants of Health, World Health Organization
Ying Tian	Shanghai Key Laboratory of Children’s Environment Health, Shanghai Jiao Tong University, China
Christopher P Wild	International Agency for Research on Cancer
Birgit Wolz	Federal Ministry for the Environment, Nature Conservation, Building and Nuclear Safety, Germany
Jun Jim Zhang	Xinhua Hospital, Shanghai Jiao Tong University School of Medicine, China

Overall, international experts stated that the JECS was a unique study that was important not only for Japan but also for the global public health community. The expected results would serve to protect and improve the health and well-being of children and generations to come throughout the world by developing legislation and social frameworks. There were two key points that the IAB highlighted during the meeting. First, by collecting data on children during the most important period of their growth, the JECS may be able to determine developmental critical windows impacted by the environment. Second, the JECS organization structure is impressive as it has led to outstanding accomplishments, including the large participant registration, high consent and response rates, and extensive collection of biological samples during pregnancy and at birth, which makes the JECS an extremely valuable birth cohort study for worldwide environmental health. The IAB recommended that the JECS should extend its originally planned follow-up period, i.e., 13 years after birth, in order to further examine the environmental impacts on adolescent health given the scarcity of such studies.

More specifically, the IAB focused on four main perspectives, i.e., JECS study design, organization structures, communication with participants and the public, and international collaboration (Table 4).

**Table 4:** Main recommendations of International Advisory Board [20]

Key points	Details of actions	Recommendations
JECS study design		
	Strategy of questionnaires	Collecting information not only from mothers but from fathers, teachers, and children themselves; appropriate frequency and length of questionnaires; use of web-based questionnaire
	Outcome measurements	Including pediatric examination for collecting objective data
	Environmental measurements	Collection of biospecimens from children; data analysis from biospecimens and questionnaires together; set priority of chemical exposures paying close attention to other relevant research projects
	Seek assent from children	Conduct a pilot study
JECS organizational structure		
	Collaboration among different disciplines	Health sector and schools
Communication with public		
	*Disseminate progress and research outcomes of JECS through media*	*Increasing presence in social media, e.g., television, newspapers, and press conferences*
	*Co-operate with public speakers*	*Seeking support from celebrities, journalists, and eminent doctors*
JECS international collaborations		
	Develop international collaboration	Sharing goals
	Share information among study teams	Study protocols, procedures, and harmonization of methodologies

*Abbreviations*: *JECS* Japan Environment and Children’s study

As for JECS study design and plans, the IAB members discussed four perspectives: (a) Questionnaires, (b) outcome measurements, (c) exposure measurements, and (d) assent acquisition from children [20]. The following were parts of discussions regarding these issues. The IAB suggested the frequency of questionnaire administration should be reduced after the participating children reach age six in order to maintain the retention rates and to reduce the participants’ burden. The participants may feel tired from too many questionnaires and lose motivation to continue their engagement. On the contrary, to assess the within-individual variation in environmental exposures, intervals of 6 months between questionnaires might be too long. More frequently applied shorter questionnaires may be beneficial for assessment of some exposure sources such as food consumption and time-location-patterns, which are subject to recall bias. Web-based questionnaires could also be used in collecting information from children. In Japan, since most parents and guardians use mobile phones, the questionnaires could be adapted for mobile use as well. Electronic questionnaires such as web-based ones can also be designed in a way that the participating children would feel as if they are playing a game.

The IAB members recommended JECS to conduct examinations by pediatricians on as many participating children as possible. Since a nationwide disease registry system is not available in Japan, the planned pediatric examinations would be important for improving the quality of children’s health assessment.

The IAB’s suggestion regarding the assessment of environmental exposure was to collect biospecimens from children around 6 years old. Biological samples from children will be important for directly assessing children’s exposure levels. Data from biomonitoring and questionnaires should be evaluated together. Long-term storage of biospecimens is important for later analysis. JECS should pay close attention to other relevant research projects in the selection and measurements of priority contaminants. Collaborations with other international research projects would be helpful in selecting the target chemical substances for the study and avoiding unnecessary effort to develop new methods for measurements and analyses.

Finally, the IAB recommended that JECS should carefully study the need and timing of obtaining informed consent/assent from participating children. A pilot trial should be conducted to ensure that this process does not jeopardize the long-term participation of the children. The age at which they will be asked for their assent or consent to the study should be defined after thorough consideration. Experiences in European cohort studies could be helpful for the JECS. They obtained informed consent from children at 18 years of age.

The second perspective reviewed by the IAB was the JECS organizational structure. The IAB recommends more intensive collaboration with the health sector of the government, including the Ministry of Health, Labour and Welfare, for the further progress of JECS. Although JECS is a MOE project with a focus on the effects of chemical exposure on children’s health, the JECS outcomes can be applicable to the development of the public health practice in much broader areas. The involvement of the Ministry of Health, Labour and Welfare would have a wide range of positive effects on the study quality of JECS as well as the development of the children’s health policy. MOE was recommended to cooperate with other ministries and agencies within the Japanese government to further utilize JECS outcomes. It would be beneficial for JECS to cooperate with school teachers and educational institutions in evaluating children’s behavior, activities, and development, because the participating children spend long hours at school once they enter. The IAB also recommended that JECS collaborate with other research projects, professionals, and medical communities.

The third perspective of the IAB review was communication with the public as well as participants to make JECS more visible, which would be important for them to recognize the value of maintaining their support for the study. Such communication efforts are essential to pursue research outcomes of high quality. The JECS presence in social media through the community is also critical and important. Press conferences and support from celebrities, specialized journalists, international organizations, and non-government organizations should also be explored further. The appearance of JECS representatives on television and in newspapers is effective as well.

Fourth, the IAB recommended that the JECS should lead international collaboration and promote more recognition from the international communities. This recommendation was based on their understanding that JECS had been involved in activities of the Environment and Child Health International Birth Cohort Group (ECHIBCG) [21], which have developed a framework on harmonization of exposure and outcome measurements between large-scale studies as described in the next section. Since JECS and other birth cohort studies share common goals to identify environmental causes of childhood diseases, collaboration with other nationwide birth cohort studies should be explored to share information about study protocols and procedures, and harmonize methodologies and instruments for analyzing biomarkers of exposure and pollutants in the environment with those studies. Such collaboration will make it possible to conduct pooled data analyses in the future.

## International collaboration with birth cohort studies and children’s environmental health projects

JECS joins several collaborative activities. JECS International Liaison Committee members have attended meetings held by international research groups on children’s environmental health. In 2011, the WHO, the United States of America, Germany, and Japan took the initiatives to hold the first meeting of the WHO Working Group on Linkage on the Next Generation of Large-scale Birth Cohort Studies in Barcelona, Spain, which was based on the recommendation made in the JECS Workshop on International Linkage and the first JECS International Symposium. Along with the United States of America, Germany, France, and Shanghai (China), JECS was a founding member of the working group that was later re-named ECHIBCG [21]. The main aims of the ECHIBCG are to share information and to work towards harmonization of processes and procedures that provide the opportunity to compare methods and results and to conduct combined analyses of results in the future. ECHIBCG members are representative researchers of large-scale birth cohorts in progress or being planned, in principle, for the sake of developing children’s environmental health. The members have held monthly telephone conferences and met in person once or twice a year to harmonize exposure/outcome measurements [21]. Until November 2016, 14 meetings of the group had been held. The recent agenda has stressed information exchange of analytical methods, quality assurance and quality control of specific chemicals, and round-robin studies of the chemicals among group members.

JECS is also a participant in the International Childhood Cancer Cohort Consortium (I4C). The I4C is an international consortium, established in 2005 to assess the association between environmental exposures and childhood leukemia [22]. Although childhood cancers impact their lives, little is known about their etiology, and their incidences are too rare to be addressed by a single study. The I4C seeks to reveal associations between prenatal and postnatal exposures and child health. Involving many cohort studies, the I4C targets up to 500,000 subjects to explore the etiology of childhood cancer.

Occasionally, JECS has received invitations and requests for international exchanges or collaborations from researchers/research teams abroad. The JECS International Liaison Committee gives close consideration to such inquiries.

## Future perspectives

The JECS international collaboration activities mentioned above will help to share information among international cohort studies and environmental health projects. Ongoing cohort studies can share their experiences on how to manage cohorts, how to develop strategies for measurements of chemical exposures, confounders, and outcomes by means of biomonitoring techniques, questionnaires, physical and development examinations.

Such international collaboration will promote replication and comparison of studies in different settings [23]. Standardizations of measurements among research teams are very important for collaboration. The ongoing collaboration will also be invaluable for analyzing differences in socioeconomic and psychosocial background among countries and regions.

## Conclusions

The JECS is expected to contribute to international environmental health research community and policy-making. More collaboration with international research teams would provide the evidence needed by policy-makers and would amplify our knowledge of ways to prevent disease and disabilities.

## Abbreviations

DNBC: Danish National Birth Cohort
ECHIBCG: Environment and Child Health International Birth Cohort Group
ELFE: Étude longitudinale française depuis l’enfance (French Longitudinal Study of Children)
I4C: International Childhood Cancer Cohort Consortium
IAB: International Advisory Board
JECS: Japan Environment and Children’s Study
MoBa: den norske Mor & barn-undersøkelsen (Norwegian Mother and Child Cohort Study)
MOE: Ministry of the Environment, Japan
